# Clinical subtypes in patients with isolated REM sleep behaviour disorder

**DOI:** 10.1038/s41531-023-00598-7

**Published:** 2023-11-17

**Authors:** Aline Seger, Anja Ophey, Christopher E. J. Doppler, Johanna Kickartz, Marie-Sophie Lindner, Maximilian Hommelsen, Gereon R. Fink, Michael Sommerauer

**Affiliations:** 1 University of Cologne, Faculty of Medicine and University Hospital Cologne, Department of Neurology, Cologne, Germany; 2 Cognitive Neuroscience, Institute of Neuroscience and Medicine (INM-3), Research Centre Jülich, Jülich, Germany; 3 University of Cologne, Faculty of Medicine and University Hospital Cologne, Medical Psychology | Neuropsychology and Gender Studies & Center for Neuropsychological Diagnostics and Interventions (CeNDI), Cologne, Germany

**Keywords:** Parkinson's disease, Parkinson's disease

## Abstract

Patients with Parkinson’s disease (PD) show a broad heterogeneity in clinical presentation, and subtypes may already arise in prodromal disease stages. Isolated REM sleep behaviour disorder (iRBD) is the most specific marker of prodromal PD, but data on clinical subtyping of patients with iRBD remain scarce. Therefore, this study aimed to identify iRBD subtypes. We conducted comprehensive clinical assessments in 66 patients with polysomnography-proven iRBD, including motor and non-motor evaluations, and applied a two-step cluster analysis. Besides, we compared iRBD clusters to matched healthy controls and related the resulting cluster solution to cortical and subcortical grey matter volumes by voxel-based morphometry analysis. We identified two distinct subtypes of patients based on olfactory function, dominant electroencephalography frequency, amount of REM sleep without atonia, depressive symptoms, disease duration, and motor functions. One iRBD cluster (Cluster I, late onset—aggressive) was characterised by higher non-motor symptom burden despite shorter disease duration than the more benign subtype (Cluster II, early onset—benign). Motor functions were comparable between the clusters. Patients from Cluster I were significantly older at iRBD onset and exhibited a widespread reduction of cortical grey matter volume compared to patients from Cluster II. In conclusion, our findings suggest the existence of clinical subtypes already in the prodromal stage of PD. Future longitudinal studies are warranted that replicate these findings and investigate the risk of the more aggressive phenotype for earlier phenoconversion and dementia development.

## Introduction

Cumulating evidence revealed a broad heterogeneity in clinical presentation and disease progression in patients with Parkinson’s disease (PD)^1^. Many attempts to identify PD subtypes have been carried out in the past, primarily focussing on motor symptoms^2^. However, PD heterogeneity may not start at disease onset—formally based on the presence of motor symptoms—but already at its prodromal stage during the incipient spread of α-synuclein aggregates^3^. This stage is characterised by the occurrence of NMS but no or only mild motor symptoms^4,5^. Hence, analysis of variation in NMS, including sleep disturbances, hyposmia, autonomic dysfunction, and cognitive impairment, might be essential for early identification of subtypes^3^.

Isolated rapid eye movement (REM) sleep behaviour disorder (iRBD) is presumed to be the most specific marker of prodromal PD^6,7^. iRBD is characterised by dream-enacting behaviours and the loss of physiological muscle atonia during REM sleep^8,9^. Longitudinal studies have demonstrated that patients with iRBD not only convert to classic ‘motor-dominant’ PD (~50% of converters), but a nearly similar proportion of patients (~45%) converts to a ‘dementia phenotype’ with relevant cognitive impairment^10,11^. In stark contrast, ~5% of patients with iRBD convert to multiple system atrophy (MSA)^10^. Thus, iRBD indicates the stage of an emerging α-synucleinopathy and represents a heterogeneous population. Additionally, considerable variability of the temporal sequence and prevalence of NMS as well as time to phenoconversion have been observed in patients with iRBD^12^.

There have been great efforts in identifying clinical biomarkers of the prodromal stage and predictors for the risk of phenoconversion: advanced age, olfactory loss, abnormal colour vision, pronounced motor symptoms, and non-use of antidepressants were identified as markers for a higher risk for short-term phenoconversion^13^. It has been shown that higher amounts of REM sleep without atonia (RWSA) point to a faster phenoconversion and to a greater risk of developing mild cognitive impairment (MCI)^11,14^. Likewise, electroencephalographic (EEG) slowing in iRBD is associated with a higher risk of developing MCI^15^. However, most studies have focused on univariate analysis using single predictors, disregarding that multiple symptoms might co-exist and yield clusters of distinct phenotypes in iRBD.

A more integrative characterisation of patients with iRBD may advance subtyping in prodromal disease stages and improve our understanding of varying disease development, helping to predict distinct disease courses, which might also be necessary for patient stratification for future clinical trials, e.g., on novel neuroprotective drugs.

We assessed motor and non-motor features, polysomnography data, and grey matter volumes in 66 patients with iRBD and conducted a two-step cluster analysis. To our knowledge, this is the first study subtyping patients with iRBD using comprehensive clinical data and cluster analysis for integrative analysis of multiple biomarkers.

## Results

We included 66 patients with iRBD (8 females) and 25 healthy control (HC) subjects (five females). The mean age of patients was 66.8 ± 6.4 years, with an average iRBD duration of 7.9 ± 6.0 years. HC subjects did not differ significantly regarding sex and age. Clinical baseline data of all patients with iRBD and HC subjects are summarised in Supplementary Table 1.

### Model characteristics of cluster analysis

Two iRBD clusters were identified by the two-step cluster analysis. Correctly identified items at Sniffin’ Sticks (variable importance index (VII): 1), dominant EEG peak frequency (VII: 0.41), disease duration (VII: 0.34), amount of RSWA (VII: 0.18), depression (VII: 0.11), and motor symptoms (VII: 0.09) were the variables with highest informative value to discriminate two clusters based on the given quality indicators. The silhouette measure of cohesion and separation was 0.3, indicating a fair structure^16^.

### Description of iRBD clusters and post-hoc comparison

Detailed characteristics of both iRBD clusters are given in Table 1 and Supplementary Table 2. The first cluster (Cluster I, *late onset*—*aggressive*) encompassed 22 patients with iRBD and was characterised by a higher age of onset of RBD symptoms but a shorter disease duration. The second cluster (Cluster II, *early onset*—*benign*) encompassed 44 patients with an earlier onset of RBD yet a longer disease duration (age at onset, Cluster I: 62.4 ± 6.2 years vs. Cluster II: 56.9 ± 7.7 years, *t*(64) = −2.935, *p* = 0.005; disease duration, Cluster I: 4.4 ± 2.6 years vs. Cluster II: 9.7 ± 6.5 years, *U* = 238.500, *z* = −3.339, *p* < 0.001). Still, both clusters of patients had comparable ages at iRBD diagnosis.

**Table 1:** Characteristics of iRBD clusters and healthy controls.

	Cluster I *n* = 22	Cluster II *n* = 44	HC *n* = 25	*P*-value
Demographic data				
Age at diagnosis (iRBD)/baseline assessment (HC) (y)	66.7 ± 6.4	66.8 ± 6.5	66.9 ± 7.6	NS
Sex (male/female)	18/4	38/6	20/5	NS
RBD-related features				
Age at onset (y)	62.4 ± 6.2	56.9 ± 7.7	–	0.005^a^
** Disease duration (y)**	4.4 ± 2.6	9.7 ± 6.5	–	**<0.001**^a^
RBDSQ	8.7 ± 3.3	9.0 ± 3.0	1.3 ± 1.5	<0.001^b,c^
Likelihood of prodromal PD (%)	95.3 ± 6.7	85.7 ± 25.5	–	NS
Parkinson’s disease motor symptom severity				
** MDS-UPDRS III**	3.5 ± 2.6	4.6 ± 2.8	–	**NS**
Autonomic function				
Orthostatic hypotension (yes/no) %	55/45	39/61	–	NS
SCOPA-AUT (total score)	7.6 ± 3.7	6.8 ± 3.6	4.1 ± 3.3	0.002^b,c^
- gastrointestinal subscore	2.0 ± 1.2	1.6 ± 1.4	0.6 ± 0.9	<0.001^b,c^
- urinary subscore	1.7 ± 0.9	1.7 ± 1.1	1.0 ± 0.9	0.040^b^
Cognition				
MoCA	27.2 ± 1.7	27.5 ± 2.0	26.6 ± 1.6	NS
Subjective Cognitive Decline	2.1 ± 1.6	0.7 ± 0.9	0.7 ± 0.8	<0.001^a,b^
** EEG peak frequency**	8.4 ± 0.7	9.5 ± 1.1	–	**<0.001**^a^
Neuropsychiatric symptoms				
** BDI-II**	8.1 ± 7.1	4.8 ± 7.1	3.3 ± 4.2	**0.011**^a,b^
BAI	5.2 ± 6.3	4.1 ± 6.3	1.8 ± 2.6	0.020^b^
FSMC	37.5 ± 17.7	29.7 ± 11.1	27.3 ± 6.6	NS
AES	30.2 ± 7.3	27.5 ± 8.1	22.7 ± 4.1	0.001^b,c^
Sleep				
** RSWA (%)**	44.4 ± 10.6	35.6 ± 15.3	–	**0.018**^a^
PDSS	12.5 ± 6.1	10.9 ± 6.5	7.3 ± 5.4	0.018^b^
ESS	5.8 ± 4.0	6.0 ± 3.2	6.2 ± 3.5	NS
Olfaction				
** Sniffin’ Sticks**	4.1 ± 2.1	7.9 ± 1.8	9.6 ± 1.6	**<0.001**^a,b,c^
General non-motor symptom burden				
NMSQ	6.8 ± 4.3	5.1 ± 3.5	2.0 ± 2.3	<0.001^b,c^

Variables included in the cluster model are highlighted in bold. *AES* Apathy Evaluation Scale; *BAI* Beck’s Anxiety Inventory; *BDI II* Beck’s Depression Inventory II; *EEG* electroencephalography; *ESS* Epworth Sleepiness Scale; *FSMC* Fatigue Scale for Motor and Cognitive Functions; *MDS-UPDRS III* Movement Disorder Society—Unified Parkinson’s Disease Rating Scale Part III, *MoCA* Montreal Cognitive Assessment; *NMSQ* Non-motor symptom questionnaire; *NS* not significant; *PDSS* Parkinson’s disease sleep scale; *RBDSQ* RBD screening questionnaire; *RSWA* REM sleep without atonia (expressed as any activity of the flexor digitorum superficialis); *SCOPA-AUT* Scales for Outcomes in Parkinson’s Disease-Autonomic questionnaire.

^a^Significant differences for pairwise comparisons between Cluster I vs. Cluster II.

^b^Significant differences for pairwise comparisons between Cluster I vs. HC.

^c^Significant differences for pairwise comparisons between Cluster II vs. HC.

**Fig. 1:**
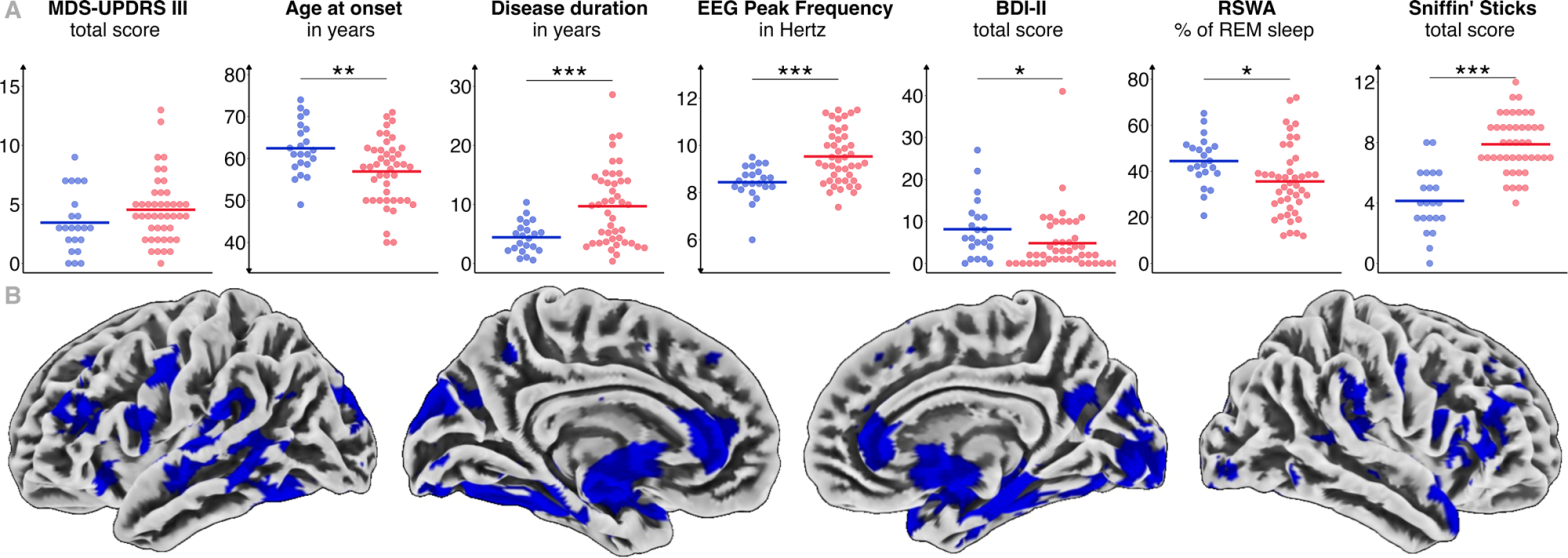
Comparison of patients with iRBD from Cluster I (*late onset*—*aggressive*) and Cluster II (*early onset*—*benign*). **A** Key clinical markers of clusters (blue = Cluster I, red = Cluster II). MDS-UPDRS III, disease duration and EEG Peak frequency were compared using Mann-Whitney U test. Age at onset and RSWA were compared using Student’s *t*-test. BDI-II and Sniffin’ Sticks were compared using Kruskal-Wallis test with post-hoc Dunn-Bonferroni. *p < 0.05, **p < 0.01, ***p < 0.001. **B** Voxel-based morphometry analysis of grey matter volume. Blue indicates reduced grey matter volume in patients from Cluster I compared to Cluster II at *p* < 0.05 (FDR-corrected). Abbreviations: BDI-II Beck Depression Inventory, EEG electroencephalography, MDS-UPDRS III Movement Disorders Society—Unified Parkinson’s disease rating scale part III, REM rapid eye movement, RSWA REM sleep without atonia.

Further, patients from Cluster I showed a higher amount of RSWA (Cluster I: 44.4 ± 10.6% vs. Cluster II: 35.6 ± 15.3%, *t*(64) = −2.431, *p* = 0.018) and slowing of EEG peak frequency (Cluster I: 8.4 ± 0.7 Hz vs. Cluster II: 9.5 ± 1.1 Hz, *U* = 221.500, *z* = − 3.576, *p* < 0.001). Besides, patients of Cluster I experienced subjective cognitive decline (SCD) in more cognitive domains (Cluster I: 2.1 ± 1.6 vs. Cluster II: 0.7 ± 0.9, *H*(2) = 15.378, *z* = 3.731, *p* = 0.001), although no difference in the Montreal Cognitive Assessment (MoCA) testing was observed (Cluster I: 27.2 ± 1.7 vs. Cluster II: 27.5 ± 2.0). They showed a higher burden of depressive symptoms (Beck Depression Inventory-II (BDI-II), Cluster I: 8.1 ± 7.1 vs. Cluster II: 4.8 ± 7.1, *H*(2) = 9.095, *z* = -2.439, *p* = 0.044) and hyposmia (Sniffin’ Sticks, Cluster I: 4.1 ± 2.1 vs. Cluster II: 7.9 ± 1.8, *H*(2) = 46.587, *z* = 4.772, *p* < 0.001) (Fig. 1). Within Cluster I, three subjects took antidepressive medication, in Cluster II, this was the case for two subjects. The clusters did not significantly differ regarding motor symptoms, orthostatic blood pressure dysregulation, subjective sleep disturbances, anxious symptoms, and non-motor symptoms as assessed with the non-motor symptom questionnaire (NMSQ).

Voxel-based morphometry (VBM) analysis revealed a more widespread grey matter volume loss in patients from Cluster I compared to patients from Cluster II with significantly lower grey matter volume in the occipital, frontal and temporal lobes, the caudate nucleus, and the cerebellum (Fig. 1).

Similar to the between-cluster analysis, patients from Cluster I showed a higher burden of depressive symptoms and declared SCD more frequently than HC subjects. Additionally, Cluster I patients suffered more frequently from anxiety symptoms and subjective sleep disturbances than HC subjects. Both clusters of patients with iRBD significantly differed from HC subjects in RBD screening questionnaire (RBDSQ), Apathy Evaluation Scale (AES), and NMSQ scores as well as in olfactory function.

## Discussion

In this study, using comprehensive clinical phenotyping and cluster analysis, we could identify two distinct subtypes of patients with iRBD. One subgroup of patients (Cluster I) was characterised by a *late onset*—*aggressive* phenotype: despite comparable age at diagnosis and motor symptom burden, these patients had a shorter disease duration, older age at RBD symptom onset, higher RSWA amount, EEG slowing, pronounced hyposmia, and accentuated neuropsychiatric symptoms including SCD and depressive symptoms compared to patients with iRBD from the *early onset*—*benign* subtype (Cluster II). Additionally, patients from Cluster I exhibited a more widespread decrease in grey matter volume compared to Cluster II.

Notably, a nearly equal proportion of patients with iRBD convert to a dementia-dominant phenotype and classic, motor-predominant PD. Even though, we cannot predict the outcome of phenoconversion in our sample due to the cross-sectional design of our study, the different clusters might represent corresponding different prodromal disease subtypes^7,17^. Recently, operationalised prodromal criteria for DLB were published and proposed biomarkers, besides iRBD, were EEG slowing, cortical grey matter volume loss, and the occurrence of neuropsychiatric symptoms—all of these biomarkers were features of Cluster I^17^. Moreover, a recent study revealed that cortical grey matter loss in patients with iRBD was linked to a greater risk of developing MCI^18^. Patients from Cluster I reported SCD in more cognitive domains than Cluster II. SCD is presumed to be an intermediate state between age-appropriate cognition and MCI. Thus, SCD is considered a risk factor for developing dementia^19–21^. Hence, patients from Cluster I are likely to be at higher risk of developing DLB or PD-D. Depressive symptoms may impact the experience of SCD^22^; however, mean BDI-II total scores of patients from both clusters did not reach the proposed cut-off of 9 as an indicator of mild depression^23^.

Interestingly, post-mortem studies have suggested that the extent of olfactory impairment is not correlated to α-synuclein pathology in the olfactory bulb but with a more general and widespread cortical and subcortical α-synuclein pathology^24,25^. This observation fits well with our findings that patients of Cluster I had pronounced olfactory impairment, which was the variable with the highest informative value to discriminate the two clusters of patients. Moreover, patients from Cluster I also exhibited reduced cortical grey matter volume. Secondly, our finding on age-related subtypes aligns with previous reports in patients with PD, proposing a higher age at disease onset to predict a more aggressive PD phenotype^26,27^. Ageing, in general, is one of the most significant risk factors for developing PD^28^, and its influence on disease-related factors, such as genetic variants, is well established^29^. Despite this epidemiological evidence, the interplay between ageing effects and neurodegenerative processes is still poorly understood. In general, the manifold clinical presentation and disease course of PD may rely on multiple individual factors independent of age^30,31^. Yet, the cause of a potential interindividual or tissue-specific vulnerability remains unclear. Aside from host-specific factors, specific α-synuclein strains may contribute to the diverse clinical phenotypes^32^. To add more complexity, additional neuropathological changes, i.e., amyloid aggregates, might add to the α-synuclein pathology, and the molecular structures of α-synuclein aggregates might not only differ between individuals but also across brain areas^32–34^.

Surprisingly, emerging motor symptoms were the least relevant factor within our cluster solution. This finding may be attributed to the circumstances that patients with iRBD express only mild motor symptoms, which are hardly differentiated by the Movement Disorder Society—Unified Parkinson’s Disease Rating Scale Part III (MDS-UPDRS III) due to a floor effect of the scale, potentially hampering the detection of subtle differences between groups. More sensitive motor assessments might have elucidated differences between the subgroups^35^. Conversely, our findings emphasise the importance of including NMS in subtyping α-synucleinopathies, particularly in the early stages.

Our study has several limitations and strengths. Most importantly, we only used cross-sectional data as longitudinal follow-up was yet unavailable. Hence, the impact of the detected subtypes on the future disease course remains speculative and it is possible that our assumptions may not be confirmed. It should be taken into consideration that the clusters may to some point represent different stages of the disease, e.g. subjects of cluster I may be closer to phenoconversion^36^. Additionally, some patients with iRBD might not convert at all due to misdiagnosis or an extraordinary benign course of the disease. However, this proportion might be rather small as a recent meta-analysis estimated that >95% of patients with iRBD phenoconvert eventually. We also included subjects with younger age at anticipated iRBD onset. Although patients with iRBD with younger age of onset may have a lower likelihood of having a neurodegenerative aetiology of RBD, additional investigations, e.g. olfactory performance, DaTSCAN imaging or skin biopsies, strengthened the likelihood of an underlying synucleinopathy in these subjects (Supplementary Table 3). As soon as longitudinal data is available in our cohort, we will evaluate the impact of the subtypes identified by this study on disease progression. However, despite the lack of longitudinal data, we observed a significantly shorter self-reported symptom duration in the *late onset*—*aggressive* cluster in the present analysis. Nevertheless, self-reported symptom duration might be biased by the awareness of a subject as there are no objective markers available to collect information about age at disease onset and disease duration at this point. One strength of the applied cluster analysis is that we used data from multiple clinically relevant domains. Applying a similar cluster analysis approach in existing, deeply-phenotyped, longitudinal iRBD cohorts would be of high value to validate our findings. It must be noted that the outcome of each cluster solution highly depends on the variables included in the model. Therefore, we aimed to include one objectively assessed biomarker for a variety of motor and non-motor categories to avoid highly correlating markers within the same category, and followed Mestre et al.’s recommendations on reporting the subtyping approach^37^. Still, neuropsychiatric symptoms mainly were assessed through questionnaires which depend on the individuals’ subjective perception and the extend of neuropsychiatric symptoms may have been biased by the recruitment via newspaper advertisement (e.g., subjects with major depression or pronounced cognitive decline are less likely to actively respond to an advertisement).

In conclusion, this study demonstrated distinct clinical subtypes in patients with iRBD, elucidating relevant differences in the expression of symptoms and potential disease trajectories primarily based on non-motor assessments. We are convinced that subtyping patients within the prodromal stage will improve the understanding of the underlying pathological pathways and hopefully help guide therapeutic decisions in the near future.

## Methods

### Participants

Participating patients with iRBD were recruited from our local iRBD cohort at the University Hospital Cologne^38^. The cohort was consecutively recruited from the general population by newspaper advertisements including the German version of the single-question screen for RBD (RBD1Q) followed by a structured telephone screening. Inclusion criteria for full screening was the answer “yes” to the RBD1Q. The structured telephone screening included demographic data, medical history and sleep questionnaires (RBD screening questionnaire (RBDSQ), Pittsburgh Sleep Quality Index (PSQI), STOP-Bang questionnaire, Epworth Sleepiness Scale (ESS), Regensburg Insomnia Scale (RIS), and screening for Restless-Legs-Syndrom). Exclusion criteria for full screening were any known neurological disorder, age <35 years or >80 years, early age of symptom onset (<35 years), alcohol or drug abuse, and having a pacemaker^38^. Based on information of the telephone screening selected subjects were invited to video-polysomnography (PSG). All candidates were asked to stop antidepressive medication 2 weeks before PSG (*n* = 5). Subjects diagnosed with iRBD according to the International Classification of Sleep Disorders III criteria for RBD^39^ were invited for a clinical assessment and underwent MRI scan.

For the current cluster analysis, we only included patients with (self-reported) age at onset over 40 years. Furthermore, the current analysis only included patients with a completed clinical assessment. In self-evaluation questionnaire data, this was defined as at least 80% valid data within a questionnaire. If an entire questionnaire was missing, subjects were excluded. For comparison, we included clinical datasets of 25 matched HC subjects without a known movement or sleep disorder who participated in independent studies at the Department of Neurology of the University Hospital Cologne. The local ethic committee of the Medical Faculty of the University of Cologne approved the study. All participants gave written informed consent before participation.

### Clinical assessment

Assessment of patients with iRBD included the collection of disease-related features, PSG data, non-motor and motor testing, and self-evaluation questionnaires from different categories:

Demographic data

- Age at diagnosis: patient’s age at the date of PSG execution
- Sex

RBD-related features

- Age at onset: age at the self-reported first occurrence of dream-enacting behaviour
- Disease duration: duration between age at onset and age at diagnosis
- RBD symptoms: RBDSQ range 0–13 (a higher score indicates a higher probability of suffering from RBD)^40^
- Likelihood of prodromal PD: according to the MDS research criteria for prodromal PD (range 0–100%, subjects with 80% or higher are considered to have prodromal PD)^6,7^

PD motor symptom severity

- Motor performance: MDS—UPDRS III (range 0–132, higher scores indicate increased motor symptom severity)^41^

Autonomic function

- Orthostatic testing: Blood pressure was assessed twice between 8 to 10 min of lying supine and after 3 min of consecutive standing. According to the consensus criteria, we considered orthostatic hypotension (OH) as a singular systolic blood pressure drop ≥20 mmHg or a diastolic blood pressure drop ≥10 mmHg upon standing compared to any supine blood pressure measurement^42^.
- Self-reported autonomic symptoms: Scales for Outcomes in Parkinson’s Disease-Autonomic questionnaire (SCOPA-AUT, range 0–63, higher scores indicate higher autonomic symptom burden)^43^. Besides the sum score, we calculated subscores for gastrointestinal function (question 1–7) and urinary symptoms (question 8–13).

Cognition

- Cognitive screening: MoCA (range 0–30, a score <26 was considered positive for MCI screening)^44^
- Subjective Cognitive Decline: Based on previous SCD assessments, SCD was assessed with dichotomous “yes or no” questions (e.g., “Do you feel like your memory is becoming worse?”) concerning six cognitive functions (memory, attention, language, executive functions, visuo-spatial skills, and social cognition)^22,45^. For further analysis, the sum score of subjectively impaired cognitive domains was calculated (0–6 points, one point per domain).
- Electroencephalography dominant frequency: EEG was recorded in the evening of the PSG in a wake, resting-state condition. The frequency with the highest power on leads O1 and O2 according to the 10–20 system in a spectrum of 6–13 Hz was identified as the dominant frequency.

Neuropsychiatric symptoms

- Depression: BDI—II (range 0–63, higher scores indicate higher symptom burden)^23^
- Anxiety: Beck Anxiety Inventory (BAI, range 0–63, higher scores indicate higher symptom burden)^46^
- Fatigue: Fatigue Scale for Motor and Cognitive Functions (FSMC, range 10–100, higher scores indicate higher symptom burden)^47^
- Apathy: AES (range 0–54, higher scores indicate higher symptom burden)^48^

Sleep

- Amount of RSWA: expressed as any activity of the flexor digitorum superficialis (FDS) muscles during overnight PSG according to the SINBAR scoring scheme assessed with *RBDtector* software^9^
- Sleep disturbances: Parkinson’s disease sleep scale (PDSS, range 0–60, higher scores indicate higher symptom burden)^49^
- Daytime sleepiness: Epworth Sleepiness Scale (ESS, range 0–24, daytime sleepiness is supposed if the sum score is ≥10)^50^

Olfaction

- Hyposmia: Sniffin‘ Sticks (range 0–12, hyposmia was defined as identification of <10 sticks)

General non-motor symptom burden

- Self-reported non-motor symptoms: NMSQ (range 0–30, higher scores indicate higher non-motor symptom burden)^51^

Assessments of HC subjects included self-evaluation questionnaires (BDI-II, BAI, FSMC, AES, NMSQ, SCOPA-AUT, ESS, PDSS), olfactory testing (Sniffin‘ Sticks) and cognitive assessment (MoCA). HC subjects did not undergo PSG, orthostatic testing, motor examination, or magnetic resonance imaging (MRI).

### Image acquisition and preprocessing

MRI measurements were obtained during clinical routine using a 1.5 T Siemens MR scanner (Siemens, Erlangen, Germany) at the Department of Radiology, University Hospital Cologne. T1-weighted brain images of 57 iRBD patients were collected and acquired using a magnetisation-prepared rapid acquisition with gradient-echo (MP-RAGE) sequence with the following parameters: 7.6 ms repetition time, 3.5 ms echo time, 8 degree flip angle, 150 slices, 266 × 246 × 142 mm field of view, 280 × 216 matrix resolution (voxel size: 0.95 × 0.95 × 0.95 mm3). Data were preprocessed and analysed using the CAT12 toolbox (https://neuro-jena.github.io/cat/). Images were reoriented and aligned to the anterior commissure, followed by segmentation into grey matter (GM), white matter (WM), and cerebrospinal fluid (CSF). The resulting images were normalised to the Montreal Neurological Institute (MNI) space, modulated using the Jacobian determinant, and smoothed using a Gaussian kernel with a value of 8 mm full width at half maximum.

### Statistical analysis

Statistical analyses were performed using SPSS 28.0. Single missing values were imputed by group-wise (iRBD vs. HC) means. We carried out a two-step cluster analysis allowing a balanced inclusion of categorical and continuous variables. Continuous variables were z-standardised (*M* = 0, SD = 1) to unify the range of their values. Cluster solutions based on 1 to 15 clusters were compared and the most suitable solution (i.e., the number of clusters) was selected according to the lowest Akaike’s Information Criterion (AIC) evaluated across cluster solutions. A variable importance index as implemented in SPSS with a range from 0 to 1, with 1 indicating the highest importance, was reported for each variable contributing to the final cluster solution. As the resulting clustering is highly dependent on the entered variables, we aimed to include one objective biomarker (e.g., RSWA instead of RBDSQ) of each category to avoid highly correlating variables of the same category (for categories, see “Clinical Assessment”). Different combinations were applied to the two-step clustering algorithm, and finally, the best solution was chosen based on quality indicators (i.e., silhouette measure of cohesion and separation, and AIC). Additional cluster solutions are presented in Supplementary Fig. 1.

Data were checked for normal distribution using the Shapiro Wilk test and the Kolmogorov Smirnov test. Chi-square-tests, one-way ANOVA, Kruskal-Wallis tests with post hoc Dunn-Bonferroni, and Student’s *t*- or Mann–Whitney *U*-tests were performed for comparisons between resulting clusters and HC subjects, as appropriate. If not stated otherwise, data are presented as mean value ± standard deviation. *P*-values < 0.05 were considered significant.

To analyse differences in VBM, we compared the smoothed GM images of the two groups resulting from the cluster analysis (Cluster I, *n* = 18; Cluster II, *n* = 39) using a two-sample *t*-test. Total intracranial volume, calculated using the CAT12 toolbox, was included as a covariate to correct for differences in brain sizes. The resulting second-level model was analysed using a non-parametric permutation test with 5000 permutations performed by the TFCE (threshold-free cluster enhancement) toolbox included in CAT12. The statistical significance threshold was set to *p* < 0.05 (FDR-corrected).

### Reporting summary

Further information on research design is available in the Nature Research Reporting Summary linked to this article.

### Supplementary information

Supplemental material

Reporting summary checklist

## Data Availability

The datasets used during the current study are available from the corresponding author on request. The data are not publicly available due to the inclusion of information that could compromise the participants’ privacy.
