# Supplementary material for: Clinical subtypes in patients with isolated REM sleep behaviour disorder
Source: NPJ Parkinsons Dis. 2023 Nov 17;9:155. doi: 10.1038/s41531-023-00598-7 (PMC10656506; doi:10.1038/s41531-023-00598-7)
Supplement: Supplementary file 1 — Supplemental material [file 41531_2023_598_MOESM1_ESM.pdf]

Supplementary material

Supplementary Table 1 Characteristics of all iRBD patients and healthy controls

	**iRBD**<br>n = 66	**HC**<br>n = 25	***P* value**
Demographic data			
Age at diagnosis (iRBD)/baseline assessment (HC) (y)	66.8 ± 6.4	66.9 ± 7.6	NS
Sex (male/female)	56/10	20/5	NS
RBD-related features			
Age at onset (y)	58.4 ± 7.6	-	-
**Disease duration (y)**	8.0 ± 6.0	-	-
RBDSQ	8.9 ± 3.1	1.3 ± 1.5	< .001
Likelihood of prodromal PD (%)	88.9 ± 21.6	-	-
Parkinson's disease motor symptom severity			
**MDS-UPDRS III**	4.2 ± 2.7	-	-
Autonomic function			
Orthostatic hypotension (yes/no) %	44/56	-	-
SCOPA – AUT (total score)	7.1 ± 3.7	4.1 ± 3.3	< .001
- gastrointestinal subscore	1.7 ± 1.3	0.6 ± 0.9	< .001
- urinary subscore	1.7 ± 1.0	1.0 ± 0.9	.009
Cognition			
MoCA	27.4 ± 1.9	26.6 ± 1.6	.060
Subjective Cognitive Decline	1.2 ± 1.3	0.7 ± 0.8	NS
**EEG peak frequency**	9.2 ± 1.1	-	-
Neuropsychiatric symptoms			
**BDI-II**	6.0 ± 7.2	3.3 ± 4.2	.090
BAI	4.5 ± 6.3	1.8 ± 2.6	.043
FSMC	32.3 ± 14.0	27.3 ± 6.6	.089
AES	28.4 ± 8.0	22.7 ± 4.1	<.001
Sleep			
**RSWA (%)**	38.5 ± 14.5	-	-
PDSS	11.5 ± 6.4	7.3 ± 5.4	.006
ESS	5.9 ± 3.5	6.2 ± 3.5	NS
Olfaction			
**Sniffin' Sticks**	6.6 ± 2.6	9.6 ± 1.6	< .001
General non-motor symptom burden			
NMSQ	5.7 ± 3.8	2.0 ± 2.3	< .001

Variables included in the cluster model are highlighted in bold.

AES Apathy Evaluation Scale; *BAI* Beck's Anxiety Inventory; *BDI-II* Beck's Depression Inventory II; *EEG* electroencephalography; *ESS* Epworth Sleepiness Scale; *FSMC* Fatigue Scale for Motor and Cognitive Functions; *MDS-UPDRS III* Movement Disorder Society - Unified Parkinson's Disease Rating Scale Part III; *MoCA* Montreal Cognitive Assessment; *NMSQ* Non-motor symptom questionnaire; *NS* not significant; *PDSS* Parkinson's disease sleep scale; *RBDSQ* RBD screening questionnaire; *RSWA* REM sleep without atonia (expressed as any activity of the flexor digitorum superficialis); *SCOPA-AUT* Scales for Outcomes in Parkinson's Disease-Autonomic questionnaire.

Supplementary Table 2

		Cluster I n = 22	Cluster II n = 44	HC n = 25	*P* value[a]	*P* value[b]
**RBDSQ**						
-	RBD positive (≥ 5 points)	86.4 % (19)	90.9 % (40)	4 % (1)	< .001	NS
**MDS-UPDRS III**						
-	subthreshold parkinsonism (> 6 points)	18.2 % (4)	13.6 % (6)	N/A	NS	
**MoCa**						
-	mild cognitive impairment (< 26 points)	18.2 % (4)	11.4 % (5)	24.0 % (6)	NS	
**BDI-II**						
-	mild depression (14-19 points)	9.1 % (2)	2.3 % (1)	4 % (1)	NS	
-	moderate depression (20-28 points)	9.1 % (2)	0 % (0)	0 % (0)		
-	major depression (> 29 points)	0 % (0)	2.3 % (1)	0 % (0)		
**EEG peak frequency**						
-	EEG slowing (< 8 Hz)	13.6 % (3)	2.3 % (1)	N/A	NS	
**BAI**						
-	mild anxiety (8-15 points)	13.6 % (3)	4.5 % (2)	4 % (1)	NS	
-	moderate anxiety (16-25 points)	9.1 % (2)	4.5 % (2)	0 % (0)		
-	severe anxiety (>26 points)	0 % (0)	2.3 % (1)	0 % (0)		
**FSMC**						
-	mild fatigue (> 42 points)	4.4 % (1)	4.5 % (2)	0 % (0)	NS	
-	moderate fatigue (> 52 points)	9.1 % (2)	0 % (0)	0 % (0)		
-	heavy fatigue (> 62 points)	13.6 % (3)	4.5 % (2)	0 % (0)		
**AES**						
-	apathy (≥ 33 points)	50 % (11)	29.5 % (13)	4 % (1)	< .001	NS
**PDSS**						
-	clinically relevant sleeping disorder (≥ 18 points)	18.2 % (4)	22.7 % (10)	8.0 % (2)	NS	
**ESS**						
-	excessive daytime sleepiness (≥ 10 points)	18.2 % (4)	13.6 % (6)	16.0 % (4)	NS	
**Sniffin Sticks**						
-	hyposmia (7-9 points)	9.1 % (2)	59.1 % (26)	32.0 % (8)	< .001	< .001
-	anosmia (≤ 6 points)	90.9 % (20)	20.5 % (9)	4 % (1)		
**NMSQ**						
-	mild symptoms (< 10 points)	77.3 % (17)	84.1 % (37)	100 % (25)	NS	
-	moderate symptoms (10 – 20 points)	22.7 % (5)	15.9 % (7)	0 % (0)		
-	heavy symptoms (> 20)	0 % (0)	0 % (0)	0 % (0)		

[a] Comparison between Cluster I, Cluster II, and HC. [b] Comparison between Cluster I vs. Cluster II.

AES Apathy Evaluation Scale; *BAI* Beck's Anxiety Inventory; *BDI II* Beck's Depression Inventory II; *EEG* electroencephalography; *ESS* Epworth Sleepiness Scale; *FSMC* Fatigue Scale for Motor and Cognitive Functions; *MDS-UPDRS III* Movement Disorder Society - Unified Parkinson's Disease Rating Scale Part III, *MoCA* Montreal Cognitive Assessment; *NMSQ* Non-motor symptom questionnaire; *PDSS* Parkinson's disease sleep scale; *RBDSQ* RBD screening questionnaire; *SCOPA-AUT* Scales for Outcomes in Parkinson's Disease-Autonomic questionnaire.

Statistics: Pearson chi-quadrat test was used for comparison between Cluster I, Cluster II, and HC. If significant, pearson chi-quadrat test was applied to test for significant differences between Cluster I and Cluster II.

Additional characteristics of subjects with age at onset ≤ 50 years

iRBD subjects with a younger age of onset have a lower likelihood of having a neurodegenerative etiology. However, cut-off values for age of onset to certainly exclude a neurodegenerative process in patients with iRBD do not exist. As manifest PD is often diagnosed from 55 years of age upwards and it is suggested that the prodromal phase can take up to 20 years, younger subjects were included in this study to not loose sensitivity. Results of additional investigations strengthened an underlying synucleinopathy in the included patients with age at onset below 50 years:

Supplementary Table 3 Additional characteristics of subjects with age at onset ≤ 50 years

Subject	***Age of Onset (years)***	***Disease Duration (years)***	***Pathologic DaTSCAN***	***Positive skin biopsy (a-syn)*** [a]	***Positive family history***	***Hyposmia***	***Likelihood prodromal PD*** [b]
**A**	40	21.4					71.8 %
**B**	40	15.3		X			24.4 %
**C**	42	15.3				X	89.1 %
**D**	47.5	15.2	X				99.5 %
**E**	48	10			X	X	99.3 %
**F**	49	5.1	X			X	99.9 %
**G**	49	20.1	X	X		X	99.9 %
**H**	50	17.4				X	97.2 %
**I**	50	28.6		X	X	X	99.5 %
**J**	50	21.6			X	X	94.7 %
**K**	50	17.3				X	99.9 %
**L**	50	13.7				X	73.5 %
**M**	50	10	X	X		X	99.9 %
**N**	50	5.7		X			87.0 %

X = the characteristic applies.

[a]Conducted within this study: Kuzkina A, Panzer C, Seger A, et al. Dermal Real-Time Quaking-Induced Conversion Is a Sensitive Marker to Confirm Isolated Rapid Eye Movement Sleep Behavior Disorder as an Early α-Synucleinopathy. Mov Disord. 2023 Jun;38(6):1077-1082. doi: 10.1002/mds.29340.

[b]Heinzel S, Berg D, Gasser T, et al. Update of the MDS research criteria for prodromal Parkinson's disease. Mov Disord 2019; 34(10):1464–70. https://doi.org/10.1002/mds.27802.

Additional cluster solutions

In Supplementary Figure 1 we visualized additional cluster solutions. Included variables for each cluster solution are framed in bold. For practical reasons, we defined Cluster I as the smaller cluster and Cluster II as the larger cluster within each cluster solution. We calculated the mean value for each variable and a red background indicates that the mean value was smaller in Cluster I than in Cluster II. Likewise, a green background means that the mean value was higher in Cluster I than in Cluster II. We aimed to include objective instead of subjective biomarker (e.g., RSWA instead of RBDSQ) and only one variable of each category to avoid highly correlating variables of the same category. The final cluster solution ("Original Cluster Solution") was additionally chosen by the AIC and Silhouette measure of cohesion and separation.

Depending on the included variables, all cluster solutions show some differences. However, across the different cluster solutions we examined, a pattern can be seen. For example, within all cluster solutions the smaller cluster included patients with a higher age at disease onset but a shorter disease duration and the second cluster included patients being younger at disease onset while having a longer disease duration. The patients from the cluster with higher age at disease onset also typically exhibited a higher burden of various non-motor symptoms.

Supplementary Figure 1 Alternative cluster solutions

	Original Cluster Solution	Cluster Solution A	Cluster Solution B	Cluster Solution C	Cluster Solution D	Cluster Solution E	Cluster Solution F	Cluster Solution G
**AIC**	280	330	320	325	390	329	686	281
**Silhouette measure of cohesion and separation**	0.3	0.3	0.3	0.3	0.3	0.3	0.3	0.3
**Distribution (Cluster 1/Cluster 2)**	22/44	32/34	18/48	32/34	28/38	29/37	27/39	21/45
**RBD related features**								
**Age at onset (y)**	*						*	
**Disease duration (y)**	*		*	*			*	
**RBDSQ (total score)**		*	*	*			*	*
**Parkinson's disease motor symptom severity**								
**MDS-UPDRS III (total score)**			*					
**Autonomic function**								
**Orthostatic hypotension (%)**					*	*		
**SCOPA – AUT (total score)**		*	*	*			*	*
Cognition								
**MoCA (total score)**		*						
**EEG peak frequency**	*							
Neuropsychiatric symptoms								
**BDI-II (total score)**	*	*	*	*			*	*
**BAI (total score)**		*	*	*			*	*
**FSMC (total score)**			*	*			*	
**AES (total score)**		*	*	*			*	*
Sleep								
**RSWA (%)**	*	*						*
**PDSS (total score)**		*	*	*			*	*
**ESS (total score)**				*			*	
Olfaction								
**Sniffin' Sticks (total score)**	*							
General non-motor symptom burden								
**NMSQ (total score)**		*	*	*			*	*

Variables included in the cluster models for each cluster solution are framed in bold. * Significant differences for pairwise comparisons between Cluster I vs. Cluster II.
Green background = Cluster I (defined as the smaller cluster) > Cluster II (defined as the larger cluster). Red background = Cluster I < Cluster II.
AES Apathy Evaluation Scale; *BAI* Beck's Anxiety Inventory; *BDI-II* Beck's Depression Inventory II; *EEG* electroencephalography; *ESS* Epworth Sleepiness Scale; *FSMC* Fatigue Scale for Motor and Cognitive Functions; *MDS-UPDRS III* Movement Disorder Society - Unified Parkinson's Disease Rating Scale Part III; *MoCA* Montreal Cognitive Assessment; *NMSQ* Non-motor symptom questionnaire; *PDSS* Parkinson's disease sleep scale; *RBDSQ* RBD screening questionnaire; *RSWA* REM sleep without atonia (expressed as any activity of the flexor digitorum superficialis); *SCOPA-AUT* Scales for Outcomes in Parkinson's Disease-Autonomic questionnaire.
