# Supplementary material for: Clinical subtypes in patients with isolated REM sleep behaviour disorder
Source: NPJ Parkinsons Dis. 2023 Nov 17;9:155. doi: 10.1038/s41531-023-00598-7 (PMC10656506; doi:10.1038/s41531-023-00598-7)
Supplement: Supplementary file 2 — Reporting summary checklist [file 41531_2023_598_MOESM2_ESM.pdf]

nature portfolio

Corresponding author(s): Michael Sommerauer

Last updated by author(s): Sep 21, 2023

Reporting Summary

Nature Portfolio wishes to improve the reproducibility of the work that we publish. This form provides structure for consistency and transparency in reporting. For further information on Nature Portfolio policies, see our Editorial Policies and the Editorial Policy Checklist.

Statistics

For all statistical analyses, confirm that the following items are present in the figure legend, table legend, main text, or Methods section.

n/a	Confirmed	
☐	☒	The exact sample size (*n*) for each experimental group/condition, given as a discrete number and unit of measurement
☐	☒	A statement on whether measurements were taken from distinct samples or whether the same sample was measured repeatedly
☐	☒	The statistical test(s) used AND whether they are one- or two-sided<br>*Only common tests should be described solely by name; describe more complex techniques in the Methods section.*
☐	☒	A description of all covariates tested
☐	☒	A description of any assumptions or corrections, such as tests of normality and adjustment for multiple comparisons
☐	☒	A full description of the statistical parameters including central tendency (e.g. means) or other basic estimates (e.g. regression coefficient) AND variation (e.g. standard deviation) or associated estimates of uncertainty (e.g. confidence intervals)
☐	☒	For null hypothesis testing, the test statistic (e.g. *F*, *t*, *r*) with confidence intervals, effect sizes, degrees of freedom and *P* value noted<br>*Give P values as exact values whenever suitable.*
☒	☐	For Bayesian analysis, information on the choice of priors and Markov chain Monte Carlo settings
☒	☐	For hierarchical and complex designs, identification of the appropriate level for tests and full reporting of outcomes
☒	☐	Estimates of effect sizes (e.g. Cohen's *d*, Pearson's *r*), indicating how they were calculated

Our web collection on statistics for biologists contains articles on many of the points above.

Software and code

Policy information about availability of computer code

Data collection	1.5 T Siemens MR scanner
Data analysis	SPSS 28.0, CAT 12 toolbox

For manuscripts utilizing custom algorithms or software that are central to the research but not yet described in published literature, software must be made available to editors and reviewers. We strongly encourage code deposition in a community repository (e.g. GitHub). See the Nature Portfolio guidelines for submitting code & software for further information.

Data

Policy information about availability of data

All manuscripts must include a data availability statement. This statement should provide the following information, where applicable:

- Accession codes, unique identifiers, or web links for publicly available datasets
- A description of any restrictions on data availability
- For clinical datasets or third party data, please ensure that the statement adheres to our policy

The datasets used during the current study are availaible from the corresponding author on request. The data are not publicly available due to the inclusion of information that could compromise the participants' privacy.

Research involving human participants, their data, or biological material

Policy information about studies with human participants or human data. See also policy information about sex, gender (identity/presentation), and sexual orientation and race, ethnicity and racism.

Reporting on sex and gender	Sex- and/or gender was based on self-reporting. No sex and/or gender-based analyses were performed due to the small sample size of female subjects. The exact group size of female and male participants are provided in the manuscript.
Reporting on race, ethnicity, or other socially relevant groupings	There were no socially constructed or socially relevant categorization variables used in our manuscript.
Population characteristics	Participants (regardless of sex, rade or ethnicity) had to be diagnosed with isolated REM Sleep Behavior Disorder (iRBD) based on over-night polysomnography. Self-reported age at onset of iRBD had to be >40 years. Subjects with manifest Parkinson's Disease were excluded. Healthy control subjects were excluded if they were diagnosed with any neurodegenerative and/or movement disorder.
Recruitment	Participants were recruited from the local iRBD cohort of the University Hospital Cologne.
Ethics oversight	Local ethic committee of the University of Cologne.

Note that full information on the approval of the study protocol must also be provided in the manuscript.

Field-specific reporting

Please select the one below that is the best fit for your research. If you are not sure, read the appropriate sections before making your selection.

☒ Life sciences ☐ Behavioural & social sciences ☐ Ecological, evolutionary & environmental sciences

For a reference copy of the document with all sections, see nature.com/documents/nr-reporting-summary-flat.pdf

Life sciences study design

All studies must disclose on these points even when the disclosure is negative.

Sample size	Subjects were recruited from our local iRBD cohort. All subjects fulfilling inclusion criteria at the point of data analyses were included in the study.
Data exclusions	Only patients with a completed clinical assessment were included. In self-evaluation questionnaire data, this was defined as at least 80% valid data within a questionnaire. If an entire questionnaire was missing, subjects were excluded.
Replication	EEG and RSWA recording was only assessed during overnight polysomnography due to the expenses of this assessement.
Randomization	Not relevant for this study.
Blinding	Not relevant for this study.

Reporting for specific materials, systems and methods

We require information from authors about some types of materials, experimental systems and methods used in many studies. Here, indicate whether each material, system or method listed is relevant to your study. If you are not sure if a list item applies to your research, read the appropriate section before selecting a response.

Materials & experimental systems

n/a	Involved in the study	
☒	☐	Antibodies
☒	☐	Eukaryotic cell lines
☒	☐	Palaeontology and archaeology
☒	☐	Animals and other organisms
☒	☐	Clinical data
☒	☐	Dual use research of concern
☒	☐	Plants

Methods

n/a	Involved in the study	
☒	☐	ChIP-seq
☒	☐	Flow cytometry
☐	☒	MRI-based neuroimaging

Magnetic resonance imaging

Experimental design

Design type	Clinical routine MRI. No task or event-related design.
Design specifications	-
Behavioral performance measures	-

Acquisition

Imaging type(s)	T1-weighted brain images (structural)
Field strength	1.5
Sequence & imaging parameters	MP-Rage, RT: 7.6 ms, ET: 3.5 ms, 8 degree flip angle, 150 slices, 266 x 246 x 142 mm field of view, 280 x 216 matrix solution (voxel size: 0.95 × 0.95 × 0.95 mm3).
Area of acquisition	Whole Brain

Diffusion MRI ☐ Used ☒ Not used

Preprocessing

Preprocessing software	CAT 12 toolbox
Normalization	Images were normalised to the Montreal Neurological Institute (MNI) space, modulated using the Jacobian determinant.
Normalization template	MNI space
Noise and artifact removal	Smoothed using Gaussian kernel with a value of 8 mm full width at half maximum.
Volume censoring	-

Statistical modeling & inference

Model type and settings	Images were reoriented and aligned to the anterior commissure, followed by segmentation into grey matter (GM), white matter (WM), and cerebrospinal fluid (CSF). Smoothed GM images (see above) of the two groups resulting from the cluster analysis were compared using a two-sample t-test. Total intracranial volume, calculated using the CAT12 toolbox, was included as a covariate to correct for differences in brain sizes. The resulting second-level model was analysed using a non-parametric permutation test with 5000 permutations performed by the TFCE (threshold-free cluster enhancement) toolbox included in CAT12.
Effect(s) tested	Group differences HC versus iRBD

Specify type of analysis: ☒ Whole brain ☐ ROI-based ☐ Both

Statistic type for inference (See Eklund et al. 2016)	Voxel-wise
Correction	Total intracranial volume, calculated using the CAT12 toolbox, was included as a covariate to correct for differences in brain sizes. The statistical significance threshold was set to $p < 0.05$ (FDR-corrected).

Models & analysis

n/a	Involved in the study
☒	☐ Functional and/or effective connectivity
☒	☐ Graph analysis
☒	☐ Multivariate modeling or predictive analysis
